# The Potential Role of Selected miRNA in Uveal Melanoma Primary Tumors as Early Biomarkers of Disease Progression

**DOI:** 10.3390/genes11030271

**Published:** 2020-03-02

**Authors:** Joanna Patrycja Wróblewska, Michał Stefan Lach, Adam Ustaszewski, Katarzyna Kulcenty, Matthew Ibbs, Inga Jagiełło, Wiktoria Maria Suchorska, Andrzej Marszałek

**Affiliations:** 1 Department of Oncologic Pathology and Prophylaxis, Poznan University of Medical Sciences, Garbary 15, 61-866 Poznan, Poland; matthew.ibbs@wco.pl (M.I.); amars@ump.edu.pl (A.M.); 2 Department of Tumor Pathology, Greater Poland Cancer Centre, Garbary 15, 61-866 Poznan, Poland; inga.jagiello@wco.pl; 3 Radiobiology Lab, Department of Medical Physics, Greater Poland Cancer, Garbary 15, 61-866 Poznan, Poland; michal.lach@wco.pl (M.S.L.); katarzyna.kulcenty@wco.pl (K.K.); wiktoria.suchorska@wco.pl (W.M.S.); 4 Department of Electroradiology, Poznan University of Medical Sciences, Garbary 15, 61-866 Poznan, Poland; 5 Institute of Human Genetics, Polish Academy of Sciences, Strzeszynska 32, 60-479 Poznan, Poland; adam.ustaszewski@igcz.poznan.pl

**Keywords:** uveal melanoma, micro RNA, metastasis, BAP1, chromosome 3 monosomy

## Abstract

Uveal melanoma (UM) is the most common primary tumor of the eye diagnosed in adults, associated with a high risk of metastasis and thereby, poor prognosis. Among known risk factors for the development of metastatic disease is the loss of BAP1 expression and chromosome 3 monosomy in the primary tumor. However, the expression levels of specific micro RNAs (miRNA) in tumor tissue may also serve as a valuable marker for determining the risk of metastatic disease in patients with primary uveal melanoma. In our study, we analyzed the miRNA expression data of cases selected from The Cancer Genome Atlas study on uveal melanoma, and determined a panel of 15 miRNAs differentially expressed between patients with primary and metastatic disease. Next, 6 miRNAs were validated on a group of 46 tumor samples from primary and metastatic patients. We have shown, that expression of hsa-miR-592, hsa-miR-346, and hsa-miR-1247 was significantly increased, while hsa-miR-506 and hsa-miR-513c were decreased in the tumors of patients with metastatic disease. Hsa-miR-196b expression did not differ between the two subgroups, however, we showed significant correlation with BAP1 expression. Moreover, hsa-miR-592 also showed correlation with monosomy 3 tumors. Gene ontology analysis revealed involvement of those miRNAs with cellular processes mediating the metastatic process. Our results showed that miRNAs play an important role in the deregulation of several oncogenic pathways in UM and can, thereby, promote metastatic spread to distant organs. Moreover, differentially expressed miRNAs may be used as an interesting biomarker for the assessment of metastatic risk in uveal melanoma patients.

## 1. Introduction

Uveal melanoma (UM) is the most common primary intraocular malignancy in adults, representing 3–5% of all melanomas. It arises mostly from choroidal melanocytes (8–90%) but can also arise from the iris (3–5%) and ciliary body (5–8%). Tumors of the ciliary body and choroid tend to be larger and more likely to form distant metastases, compared to melanomas originating from the iris. The median age of diagnosis was approximately 62 years; however, the highest peak of diagnosed cases was between 70 and 79 years. Risk factors include the presence of light-colored eyes, fair skin, inability to tan, ocular melanocytosis, and dysplastic nevus syndrome. Males have a 30% greater incidence than females. Despite the relatively good response of primary UM to treatment, which involves surgery or radiotherapy, almost 50% of patients will develop metastatic disease. The most common primary site for the development of metastasis is the liver (89%), where spread occurs only via blood vessels. The 5-year survival rate among primary disease patients is approximately 60–70%. In recurrent cases with developed metastatic disease, the median overall survival drops to approximately 13.4 months, with only 8% of patients surviving 2 years since there are no efficient therapies for metastatic UM [1,2]. Due to the high mortality rate associated with metastatic uveal melanoma, there is a need to identify molecular events driving disease development and progression in order to establish early diagnostic markers and improve existing therapeutic strategies. The Cancer Genome Atlas (TCGA) study identified some molecular features correlated with higher risk of metastasis, including chromosome 3 monosomy, loss of BRCA-associated protein (*BAP1*) expression, and eukaryotic translation initiation factor 1 (*EIFAX*), splicing factor 3 subunit 1 (*SF3B1*) mutations (increasing the risk of metastases in disomy 3 tumors) [3,4]. Alterations of G Protein Subunit α 11 *(GNA11)* and G Protein Subunit α Q *(GNAQ)*, although present in up to 90% of the tumors, do not have an impact on progression-free survival or overall survival. Other known risk factors include age, tumor size, and infiltration of the optic nerve or blood vessels [5,6]. Despite these known molecular features and the well-described molecular background of uveal melanoma development, there is no successful treatment for metastatic UM.

The TCGA study also reported another important factor that mediates UM development and progression—micro RNA (miRNA). They are a class of short (17–22 nucleotides in length), noncoding RNAs that regulate gene expression and thereby play significant roles in various pathological conditions, including cancer [7,8]. Nowadays, miRNAs are considered important biomarkers of cancer development and progression. Oncogenic miRNAs are overexpressed in cancers, while the expression of tumor-suppressive miRNAs is significantly decreased. When these particular miRNAs are inhibited or stimulated, cancer cell proliferation, metastasis, and survival may be significantly changed. However, despite several studies, the prognostic and therapeutic significance of uveal melanoma-related miRNA is still poorly understood. In this context, we aimed to validate the differential expression of miRNAs selected from the TCGA study in primary tumor tissue, and to determine their usefulness as potential early biomarkers of disease progression and targets for the development of new therapeutic strategies for metastatic uveal melanoma.

## 2. Materials and Methods 

### 2.1. Study Group

The study group consisted of 50 archived, formalin fixed, and paraffin embedded (FFPE) primary tumor samples from uveal melanoma patients, diagnosed between 2011 and 2018, retrieved from the archives of the Department of Tumor Pathology, Greater Poland Cancer Centre, Poznan, Poland. All samples were reevaluated by a pathologist, according to the 8th edition of the American Joint Committee on Cancer (AJCC) staging guidelines. Histological characteristics included tumor TNM staging. All tumors were located in the choroid, tumors from the iris were excluded from this study. Clinical data included the date of diagnosis, disease status (primary/metastatic), and time of disease progression or last follow-up. The average follow-up time was two years. Tumor samples were divided into two subgroups, based on disease status: primary and metastatic. The “primary” subgroup included primary tumor samples from patients with no known metastases by the time of the last follow-up. The “metastatic” subgroup included primary tumor samples from patients who were diagnosed with or developed metastatic disease during the follow-up period.

### 2.2. The Cancer Genome Atlas (TCGA) Data Analysis of Differentially Expressed Micro RNA

Clinical information, the miRNA-seq data of 8 patients (4 primary and 4 metastatic tumors), was collected from the TCGA Research Network (https://www.cancer.gov/tcga) and used in the current study. The raw data used for differential expression analysis were previously preprocessed with the use of the BCGSC miRNA profiling pipeline commonly used for large-scale profiling of miRNAs in the TCGA project [9]. As a result, the read counts of detected miRNAs could be obtained and used for normalization and differential expression analysis. This procedure was conducted using the Bioconductor edgeR package [10,11] with the following criteria: ≥5 counts per million (CPM) in at least two primary tumor samples and ≤2 CPM in at least two metastatic tumor samples (for selection of upregulated miRNAs in primary tumor samples) and ≥2 CPM in at least two primary tumor samples and ≤5 CPM in at least two metastatic tumor samples (for selection of upregulated miRNAs in metastatic tumor samples).

### 2.3. Gene Ontology Enrichment Analysis

To obtain the set of potential gene targets for each miRNA taken into consideration, the following freely available tools were used: TargetScan [12], miRDB [13], microT-CDS [14], and miRWalk2.0 [15]. In order to obtain sets of potential gene targets that were as accurate as possible, we performed the analysis with the use of the following criteria: Cumulative weighted context++ score ≤ −0.5 for TargetScan, Target Score ≥85 for miRDB, miTG score ≥0.95 for microT-CDS. Regarding the miRWalk2.0 tool, we chose prediction based on 12 algorithms, where the putative gene target was taken into further account only when at least 9 of the 12 algorithms resulted in positive miRNA–3′UTR interaction. Afterwards, to determine possible interactions between protein products of the obtained gene targets and to define their possible involvement in particular biological processes, we performed gene ontology (GO) analysis using the STRING database [16].

### 2.4. Tissue Microarrays

Tissue microarrays (TMA) were constructed using the semi-automated Minicore^®^ system (Mitogen, Harpenden, UK). Briefly, from each of the donor FFPE blocks, three sample cores (0.6 mm diameter) were collected and transferred to recipient TMA blocks. The tissue samples were collected in triplicate in order to reduce issues related to representativeness. A total of 270 tissue cores were included in the microarray. Subsequently, 4 µm sections from the array blocks were obtained using a microtome, and the sections were used to assess chromosome 3 status and for BAP1 expression analysis.

### 2.5. Fluorescent In Situ Hybridization (FISH) 

A fluorescent in situ hybridization (FISH) analysis was performed on 4 µm FFPE sections of the TMA blocks. After deparaffinization with xylene and ethyl alcohol, each sample was digested with proteinase solution and hybridized with fluorescently labeled Vysis CEP 3 (D3Z1) SpectrumOrange Probe (Abbott, Abbott Park, IL, USA) in an automated hybridizer (Dako Agillent, Santa Clara, CA, USA). The CEP 3 probe is designed to cover the 3p11.1–q11.1 α satellite region of chromosome 3. Cell nuclei were counterstained using DAPI solution. To determine the chromosome 3 status, 100 nuclei were counted. Results were visualized and analyzed using an Olympus microscope with fluorescence and Image Analysis Software (Olympus, Tokyo, Japan). Based on literature data, a cutoff value of 20% was used to define monosomy for chromosome 3.

### 2.6. Immunohistochemistry (IHC)

Immunohistochemical staining was performed on 4 µm FFPE sections of the TMA blocks, using the En Vision^TM^ FLEX GV800 (Dako Agillent, Santa Clara, CA, USA) IHC Kit with mouse monoclonal IgG1 antibodies for BAP1 (C-4) (Santa Cruz Biotechnology, Dallas, TX, USA). The IHC reaction was carried out on an Autostainer Link 48 system (Agillent, Santa Clara, CA, USA). Results were analyzed using an Olympus microscope (Olympus, Tokyo, Japan).

### 2.7. Quantitative Real-Time PCR

To validate differentially expressed miRNA selected from the TCGA data analysis, miRNA from primary and metastatic FFPE tumor samples was extracted with the miRNeasy FFPE Kit (Qiagen, Hilden, Germany). The complementary DNA (cDNA) was prepared from 50 ng of isolated miRNA with TaqMan™ Advanced miRNA cDNA Synthesis Kit (Applied Biosystems, Foster City, CA, USA) according to the manufacturer’s instructions.

Quantitative real-time PCR analysis was performed with TaqMan miRNA Assay specific to the chosen miRNA and TaqMan™ Universal PCR Master Mix (Applied Biosystems). U6 small nuclear snRNA was used as an internal control for the PCR reaction and as the normalization calibrator for determining relative miRNA expression levels. All real-time based analyses were performed on a Cobas z4800 device with LightCycler 480 Software (Roche, Basel, Switzerland). 

### 2.8. Statistical Analysis

All statistical analyses of the obtained results were performed in GraphPad Prism 8 (GraphPad Software, San Diego, CA, USA). Progression-free survival (PFS) was defined as the number of months from diagnosis to identification of local recurrence or metastasis. Kaplan–Meier plots and log-rank analysis were done to visually assess the differences in PFS correlated with chromosome 3 status, BAP1 and miRNA expression between subgroups. The statistical associations between clinicopathologic and immunohistochemical/FISH findings in subgroups were evaluated with the Fisher exact test. The significance of differential miRNA expression between subgroups was analyzed with the Mann–Whitney test, as the samples do not meet the criteria of the normal distribution, where * *p* < 0.05, ** *p* < 0.01, *** *p* < 0.001, **** *p* < 0.0001.

## 3. Results

### 3.1. TCGA Data Analysis of Differentially Expressed Micro RNA

To determine miRNA differentially expressed between primary and metastatic patients, we analyzed selected datasets (*n* = 8) from the TCGA Uveal Melanoma study. Samples were selected based on the presence of metastases, vital status, clinical staging, and the number of days from diagnosis to the development of metastases or death due to metastatic disease. Four patients with stage IV disease and with metastases detected around the time of diagnosis were considered to be metastatic patients. Another four patients with stage IIA disease, but without metastatic disease at the time of the last follow-up, were considered to be primary patients (Table 1). 

**Figure 1:**
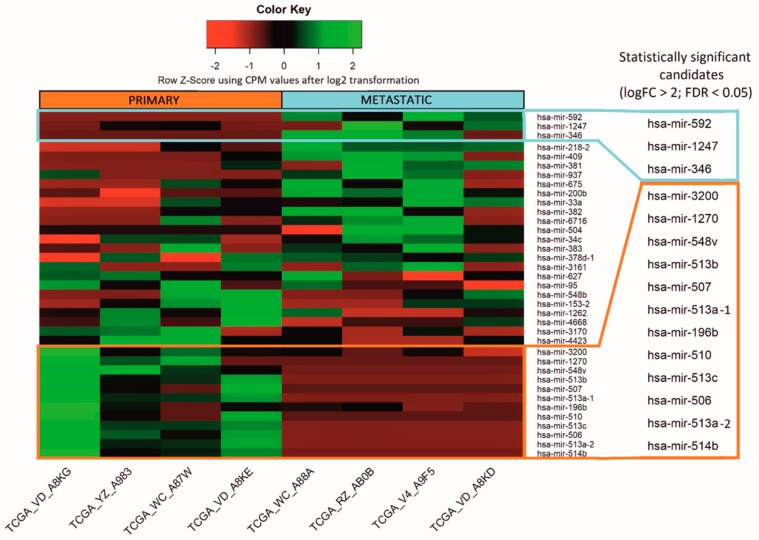
Micro RNA differentially expressed between primary and metastatic uveal melanoma TCGA samples. Altogether, the expression of 37 miRNAs differed between analyzed samples, whereas 15 were considered to be statistically significant (marked with frames), with false discovery rate (FDR) < 0.05. The results are displayed as log2 transformation of normalized CPM values.

The analysis of the miRNA expression dataset from the selected patients showed 37 differentially expressed miRNAs between primary and metastatic subgroups, of which 15 were statistically significant (false discovery rate (FDR) < 0.05 and logFC > 2). Three miRNAs were upregulated in metastatic samples and 12 in primary samples (marked with frames) (Figure 1). In order to emphasize the differences of the obtained miRNA expression profiles in the figure, we performed log2 transformation of CPM normalized values. The non-numeric values resulting from the log2 transformation of 0 (-Inf) have been replaced by zeros. The transformation was conducted only for visualization purposes.

Based on the results of the bioinformatic analysis, we selected six differentially expressed miRNAs as listed in Table 2. Three miRNAs were upregulated in metastatic tumors: hsa-mir-592, hsa-mir-1247, hsa-mir-346, and another three upregulated in primary tumors: hsa-mir-196b, hsa-mir-513c, hsa-mir-506, all with FDR below 0.05.

### 3.2. Histopathological and Molecular Characterization of Uveal Melanoma Tumor Samples

To validate the differentially expressed miRNA, we initially selected 50 primary uveal melanoma FFPE tumor samples from patients diagnosed between 2011 and 2018 in the Greater Poland Cancer Centre. Four samples were excluded from molecular analyses owing to the low quality of the tissue sample or incomplete clinical data, resulting in the final 46 patients enrolled in this study. Then, based on data from the last follow-up, samples were divided into two subgroups representing primary and metastatic samples. The first, the primary group, included 28 primary UM tumor samples from patients who still had no metastases at the time of the last follow-up. The second group was the metastatic group and included 18 primary tumor samples from patients who were reported to have metastatic disease by the time of follow-up (Table 3). 

**Figure 2:**
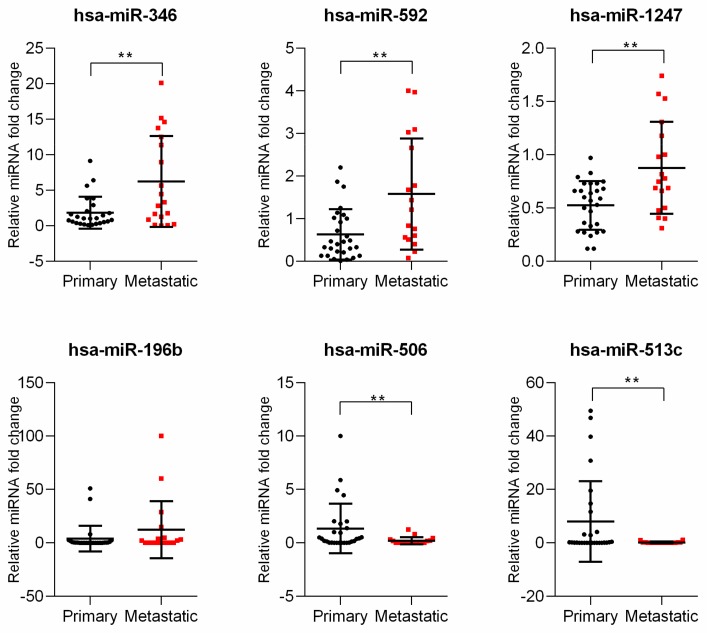
Analysis of miRNA expressed differentially between primary and metastatic uveal melanoma samples. The results are displayed as relative miRNA fold change calculated as log_2_-ΔΔCt. Each dot represents an individual patient. The graph represents mean ± SD. The statistical differences between the two groups were analyzed with Mann–Whitney test where: * *p* < 0.05, ** *p* < 0.01, *** *p* < 0.001, **** *p* < 0.0001.

The expression of six differentially expressed miRNAs were analyzed using quantitative real-time PCR in 46 uveal melanoma tumor samples. Interestingly, in these primary samples, we showed that expression of hsa-miR-592, hsa-miR-346, and hsa-miR-1247 was significantly increased in the metastatic subgroup. Moreover, we confirmed that the expression of hsa-miR-506 and hsa-miR-513c was significantly decreased in metastatic samples. Unfortunately, the expression of hsa-miR-196b showed no statistically significant differences between primary and metastatic subgroups as indicated by the bioinformatic analysis (Figure 2). 

**Figure 3:**
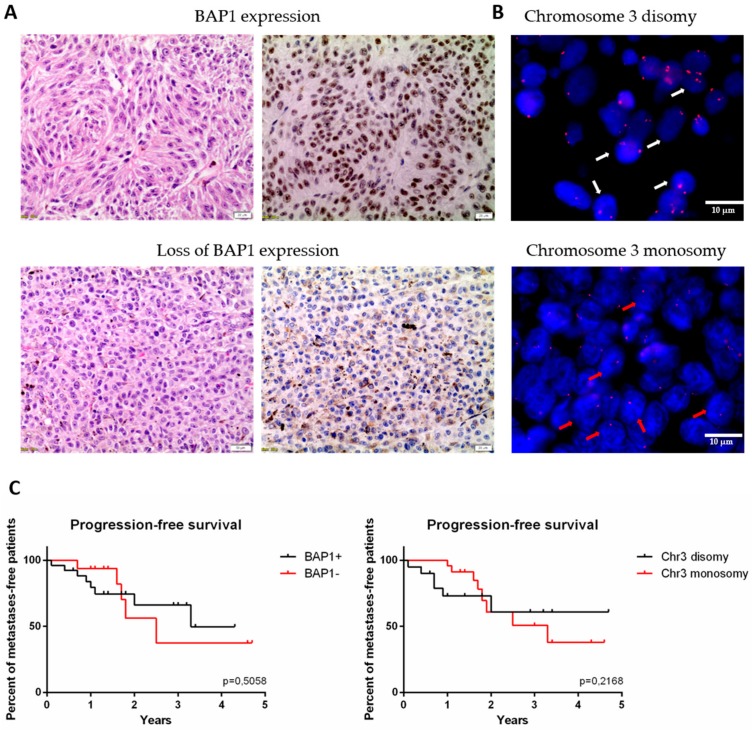
The expression of BAP1 protein and status of chromosome 3 analyzed in formalin fixed and paraffin embedded (FFPE) primary tumor samples. (**A**) The BAP1 status was analyzed with immunohistochemistry (IHC) staining. Upper panel represents tumor with BAP1 expression, bottom panel represents one with loss of BAP1 (left: hematoxylin and eosin staining; right: IHC for BAP1). Scale bar represents 20 µm, magnification: 20×. (**B**) Chromosome 3 status was analyzed with fluorescent in situ hybridization (FISH). White arrows point out the nuclei with chromosome 3 disomy, red arrows with monosomy. Scale bar represents 50 nm, magnification: 100×. (**C**) Kaplan-Meier survival curves for progression free survival in group with and without BAP1 expression (left) and monosomy 3 (right), respectively.

One of the key aspects in the development of metastasis in UM patients is the phenomenon of chromosome 3 monosomy and the loss of BAP1 expression. In order to identify correlations between selected miRNAs and the occurrence of these molecular events, we utilized tissue microarrays to perform FISH analyses to determine chromosome 3 status, and immunohistochemical staining for BAP1 expression among the UM primary tumor samples. Immunohistochemical staining showed a loss of BAP1 expression among 29 patients (60%)—17 primary and 12 metastatic (Figure 3A, Table 1). Monosomy of chromosome 3 was detected in 23 patients (50%)—14 in the primary group versus 9 in the metastatic group (Figure 3B, Table 1). Kaplan–Mayer survival analysis was performed to compare progression free survival (PFS) rates between groups defined by their chromosome 3 and BAP1^+/−^ status. Besides the finding that the PFS median was lower in the patients with detected chromosome 3 monosomy or loss of BAP1 expression, there were no statistically significant differences between the observed groups (Figure 3C). 

**Figure 4:**
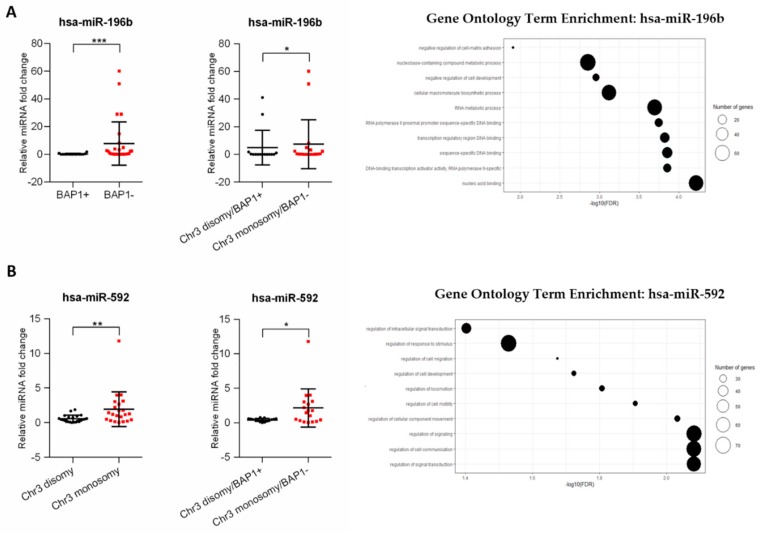
The correlation between selected miRNAs with BAP1 expression and chromosome 3 monosomy in uveal melanoma primary tumors. The results are displayed as relative miRNA fold change calculated as log_2_-ΔΔCt. The statistical differences between two groups were analyzed with the Mann–Whitney test where: * *p* < 0.05, ** *p* < 0.01, *** *p* < 0.001, **** *p* < 0.0001 (left). Gene ontology (GO) enrichment analysis for hsa-miR-196b and has-miR-592. Results are displayed as −log_10_(FDR) of given GO terms. The size of dots corresponds to the number of miRNA target genes detected in the given GO term (right). The analyses were performed for hsa-miR-196b (**A**) and hsa-miR-592 (**B**).

Correlation of analyzed miRNA with chromosome 3 status and BAP1 expression was performed. The statistical analysis showed that of six miRNAs only one was significantly increased in BAP1^-^ tumors and one in monosomy 3 tumors (Figure S1). Surprisingly, hsa-miR-196b, which expression did not differ between the primary and metastatic groups, was significantly increased in the group of tumors with loss of BAP1 expression and in the group of patients with loss of BAP1 expression and chromosome 3 monosomy (Figure 4A). Moreover, we showed that expression of hsa-miR-592 correlates with chromosome 3 status and is increased in tumors with chromosome 3 monosomy. Similarly, the expression was also significantly higher in tumors with loss of BAP1 expression and chromosome 3 monosomy (Figure 4B). To determine the functions related to the predicted target gene regulated by those miRNAs, GO enrichment analysis was performed. We showed that hsa-miR-196b target genes take part in the regulation of biological processes connected to nucleic acid metabolism, sequence-specific binding of DNA, and cell-matrix adhesion (Figure 4A). The highest number of genes regulated by hsa-miR-592 were shown to take part in the regulation of cellular signaling and signal transduction processes. Other important processes regulated by this miRNA include the regulation of cell migration and motility (Figure 4B).

Taken together, these results confirm the important role of the analyzed miRNA in the development of metastasis in uveal melanoma. Moreover, they suggest that the expression level of the analyzed miRNA, together with chromosome 3 status and BAP1 expression, may serve as a potential biomarker of disease progression and determine patients at high risk of developing metastasis. 

## 4. Discussion

In our study, we determined the set of miRNA differentiating the primary UM diagnosed patients into groups with high risk of developing metastatic disease. Moreover, some miRNAs, in correlation with chromosome 3 monosomy and BAP1 expression, may help to assess the risk of metastatic spread in UM patients. Over the past decade, miRNAs have grown to be considered a valuable diagnostic and prognostic biomarker of cancer development and progression. Recently, they are also being considered a promising therapeutic target. So far, there are no efficient targeted therapies for metastatic UM. Hence, determining of the role of the miRNA regulatory network in uveal melanoma may not only help to better understand its biology, but also in assessing the potential risk factors for disease progression, and in the development of successful therapeutic strategies for metastatic UM. Several studies have attempted to utilize miRNA expression profiling in order to point out specific prognostic miRNAs involved in the metastatic spread of uveal melanoma. TCGA consortium has determined the miRNA expression profile in numerous cancer types, including samples from uveal melanoma patients. A comprehensive analysis of 80 primary tumors identified four molecular and clinical subsets, depending on chromosome 3 status, EIF1AX, SF3B1, and BAP1 gene alteration, DNA methylation, and copy number status. The profile of miRNA expression revealed four consensus clusters that were associated with monosomy 3 tumors and their DNA methylation level [5]. Consistent with Worley’s group study, which performed microarray-based profiling of 24 fresh frozen primary uveal melanoma tumors and showed two distinctive miRNA clusters, that differentiate primary from metastatic patients, they estimated that miR-199 family and let-7b were more highly expressed in tumors which more likely will form distant metastasis [17]. Moreover, several other miRNAs, like hsa-miR-486-5p, -451a, -142, -150, -21, -29b, -146b, and -155 were also identified as highly expressed in tumors with chromosome 3 monosomy, considered to be those with higher risk of metastatic spread [5]. With all the data available to the public, TCGA data-mining has become a valuable source of comprehensive molecular datasets for identifying relationships between genetic, epigenetic alterations and clinical outcomes in cancers. Recent studies have utilized this data to identify panels of differentially expressed miRNAs with prognostic value for uveal melanoma progression. Falzone et al. determined the prognostic significance of deregulated miRNA, based on TCGA data, in a low grade vs. high-grade uveal melanoma tumor and in alive vs. deceased patient groups. They showed that among the top of 20 deregulated miRNA, seven of them were associated with tumor stage, vital status, and overall survival. Most of those miRNAs are part of a well-described miRNA-506-513 cluster, as in our study—downregulated in high-grade and deceased UM patients. The upregulation of hsa-miR-199a in this group was concordant with previous studies [18]. A similar analysis was performed by Xin et al. who used the TCGA data to determine the signature of nine miRNA for the prognosis of uveal melanoma. Among those deregulated miRNAs, hsa-miR-513c was previously reported, but otherwise, the panel showed no similarities with the set identified by Falzone et al., despite using the same source data [19]. The study of Smit et al. performed miRNA and mRNA expression profiling on 26 UM patient samples, to determine not only differentially expressed miRNA, but also their target genes and enriched cellular pathways involved in the progression of UM. The authors determined a set of 13 differentially expressed miRNAs, but in comparison with the data from this study and TCGA, some differences has been notified [20]. The discrepancies observed in those results are probably due to the use of different pipelines for next generation sequencing data analysis. The discrepancies observed in these results are probably related to usage of different pipelines during analysis of next generation sequencing data. Moreover, the study of Falzone et al., Xin et al., and TCGA consortium was based only on in silico analysis, lacking in vitro evaluation. In order to fully determine the usefulness of differentially expressed miRNA as potential biomarkers, there is the necessity for in vitro and in vivo validation of these preliminary bioinformatics data.

In our study, we utilized existing datasets of miRNA expression profiles from eight uveal melanoma patients from the TCGA database, to determine differentially expressed miRNAs between primary and metastatic patients. We selected samples for this analysis based on stage of the disease, presence and time of metastatic spread. Our analysis showed a set of 15 deregulated miRNAs of which three were upregulated in metastatic and 12 in primary patients, respectively. In order to validate six chosen miRNA, we used a cohort of 46 FFPE primary tumor samples divided onto two subgroups based on the disease status during last follow-up check: primary or metastatic UM patients. The results confirmed the preliminary in silico analysis of three miRNAs upregulated (hsa-miR-346, hsa-miR-1247, and hsa-miR-592) and two downregulated (hsa-miR-513c, miR-506) in the metastatic subgroup. Meanwhile, the hsa-miR-196b upregulation in the primary patient group during in silico analysis was not confirmed during our validation, indicating the similar level of expression in both subgroups. One of the causes could be related to the small and diverse study group. 

Two of the metastatic group-related miRNAs, hsa-miR-1247 and hsa-miR-592, have been previously described as upregulated in high-grade uveal melanoma, by Falzone et al. [18]. These results are contrary to several other studies showing that they usually act as tumor suppressors in pancreatic, colorectal, prostate, breast, and other cancers [21,22,23,24,25,26]. Similar to our studies, the overexpression of hsa-miR-592 was also described in progression of renal cell carcinoma, colorectal carcinoma with unaffected mismatch repair mechanisms, and gastric cancer [27,28,29]. In the case of hsa-miR-1247, its upregulation, contrary to our study, has shown its tumor suppressing properties in pancreatic carcinoma as well as in neuroblastoma [30,31]. These results suggest that the described miRNA may play a dual role in the development of various cancers and the determination of their exact, cancer-specific function still needs further study. Another miRNA upregulated in our metastatic patient subgroup was hsa-miR-346. For the first time, we have shown its role in uveal melanoma. Its role is important in the case of cancer biology; several studies showed its oncogenic nature in numerous cancers, which was responsible for promotion of the more aggressive phenotype of disease. It has been shown that overexpression of hsa-miR-346 promoted cell proliferation, colony formation, migration, invasion in nasopharyngeal, colorectal, and liver cancer [32,33,34,35]. Moreover, it was responsible for reduced apoptosis and resistance to docetaxel of breast cancer cells [36]

Most of the downregulated miRNAs detected in our study in the metastatic patient subgroup belong to the miRNA-506-513 cluster, which is consistent with previous TCGA-based studies [5,18]. However, their role in UM is still not a well-known phenomenon, but recent several groups indicated its engagement in the progression of the tumor. The Streicher’s group indicated that in cutaneous melanoma, the miR-506-514 cluster was consistently overexpressed and mediated the melanocytes transformation [37]. On the other hand, it has been shown that the downregulation of hsa-miR-513c promotes the proliferation and invasiveness of neuroblastoma and glioma cells [38,39]. Similar to that downregulation of hsa-miR-506, leads to increased proliferation, invasiveness, and epithelial to mesenchymal transition in breast gastric and ovarian cancer [40,41,42]. Moreover, in vitro studies showed that overexpression of this miRNA is responsible for the increased sensitivity of colorectal cancer cells to chemotherapy [42]. 

Another downregulated miRNA detected in metastatic UM patients was has-miR-196b. To the authors best knowledge, this miRNA has also not previously been described in the context of uveal melanoma progression. However, its role has been investigated in several cancers. Stiegelbauer et al. showed that low expression of hsa-miR-196b-5p was significantly associated with metastases and poor outcomes in colorectal carcinoma. Inhibition of this miRNA led to increased invasiveness of cancer cells and metastases formation in mice. [43]. Similar results were presented by Zhu et al., who showed, that high expression of miR-196b-5p was negatively associated with lymph node metastasis and the progression of the clinical stage in patients with breast cancer [44]. Considering these results, we believe that downregulation of has-miR-196b in UM, as presented in our study, may have a similar effect on uveal melanoma cells and needs further study. 

It is known that chromosome 3 monosomy and the loss of BAP1 expression are related to poor prognosis and an increased risk of developing metastatic disease in uveal melanoma [5,45,46]. Chromosome 3 and BAP1 status were also determined in our validation cohort of patients and associated with progression-free survival. We showed that average disease progression-free time was longer in patients with disomy 3 and BAP1 expression, in comparison to patients with monosomy 3 and loss of BAP1. Surprisingly though, these results were not statistically significant. Again, the reason behind this may be that an insufficient number of patients were enrolled in this study. However, we analyzed the expression levels of 6 differentially expressed miRNAs selected for validation in groups of patients defined by their chromosome 3 and BAP1 status. 

We showed that higher expression of hsa-miR-592 is associated with chromosome 3 monosomy detected in tumors. This correlation was observed by the Triozzi’ group. The authors analyzed differentially expressed miRNAs in tumors and plasma samples grouped in terms of chromosome 3 status, where hsa-miR-592 was shown to be under-expressed in monosomy 3 tumors, which contradicts our results. The association of increased has-miR-592 expression in monosomy 3 tumors and higher risk of developing metastases in this group could be explained by miRNA influence on several biological processes. We performed GO enrichment analysis for has-miR-592 target genes, and showed that this miRNA may have an impact on uveal melanoma progression through direct interaction with genes involved in cell migration, motility, development, and regulation of cellular signaling. 

In this study, we also analyzed the association between differentially expressed miRNA and BAP1 status. We showed that expression of hsa-miR-196b was significantly increased in tumors with loss of BAP1 expression. To the authors best knowledge, this is the first time this association has been presented in the context of uveal melanoma. Sharma et al. analyzed the mutational landscape of the BAP1 locus in the context of binding sites for miRNA. They showed that either germline or somatic mutations can affect the mRNA binding region located within the locus, which leads to disruption of the whole regulatory network, not only in uveal melanoma, but also in several other cancers [47]. Several different miRNAs were shown to influence BAP1 function in cervical cancer, renal carcinoma, lung cancer, breast cancer, and others [48,49,50,51]. However, hsa-miR-196b has never been previously mentioned in this context. Finally, to determine the effect of the analyzed miRNA expression on the regulation of biological processes, we performed GO analysis on the predicted target genes. We showed that hsa-miR-196b is involved in the regulation of DNA sequence-specific binding, nucleic acid binding and metabolism, regulation of transcription, and the negative regulation of cell adhesion. Most of these processes regulated by hsa-miR-196b target genes belong to the HOXA genes cluster. It has been shown that long noncoding HOXA11-AS is overexpressed in uveal melanoma cells and may act as a miRNA sponge for has-miR-124 and regulate UM cell growth, invasion, and apoptosis [52]. Described processes correlate with BAP1 function, which is protein deubiquitination as consequence participate in regulation of transcription, cell cycle, and growth, response to DNA damage and chromatin [53]. Deregulation of these processes may explain the influence of the analyzed miRNA upregulation on the increased risk of metastatic spread of uveal melanoma. 

## 5. Conclusions

In this study, we show that miRNAs play an important role in the deregulation of several oncogenic pathways in UM primary tumors and can be responsible for the promotion of metastatic spread to distant organs. Differentially expressed miRNAs are considered as an interesting biomarker for assessing metastatic risk in uveal melanoma. Moreover, until now, there are no successful treatment strategies for metastatic UM. The miRNAs expression regulation may be utilized as a promising therapeutic agent for personalized medicine. Inhibitors of miRNA expression (anti-miRs) and miRNA mimics have shown promising results in preclinical studies and could reverse the effects of dysregulated, metastasis-related cellular pathways, and thereby increase the likelihood of positive outcomes for uveal melanoma patients. Further evaluation of the inhibitors or enhancers miRNAs described in this study should be tested to indicate their therapeutic potential. 

## Figures and Tables

**Table 1:** Clinical data of The Cancer Genome Atlas (TCGA) patients selected for differential miRNA expression analysis. AJCC: American joint committee on cancer.

Patient ID	AJCC Clinical Stage	AJCC Primary Tumor (T)	Time From Pathologic Diagnosis to Follow-Up	Distant Metastasis	Time from Pathologic Diagnosis to Metastasis	Vital Status
TCGA-RZ-AB0B	Stage IV	T4b	149	Yes	0	Dead
TCGA-V4-A9F5	Stage IV	T4e	203	Yes	78	Alive
TCGA-VD-A8KD	Stage IV	T4a	114	Yes	0	Dead
TCGA-WC-A88A	Stage IV	T4d	82	Yes	0	Dead
TCGA-YZ-A983	Stage IIB	T3a	798	No	Not applicable	Alive
TCGA-VD-A8KE	Stage IIA	T2a	821	No	Not applicable	Alive
TCGA-VD-A8KG	Stage IIA	T2a	20	No	Not applicable	Alive
TCGA-WC-A87W	Stage IIA	T2a	1703	No	Not applicable	Alive

**Table 2:** FDR of differentially expressed miRNA selected for validation in tumor samples.

miRNA	FDR
hsa-mir-592	4.77 × 10^−3^
hsa-mir-1247	2.44 × 10^−2^
hsa-mir-346	2.44 × 10^−2^
hsa-mir-196b	1.44 × 10^−7^
hsa-mir-513c	9.38 × 10^−5^
hsa-mir-506	5.72 × 10^−8^

**Table 3:** The clinical, histopathological, and molecular characteristics of distinguished subgroups of studied primary tumor samples collected from uveal melanoma (UM). SD: Standard deviation.

Patients Characteristics	Primary, *n* = 28	Metastatic, *n* = 18
Age of diagnosis		
Mean ± SD	63 ± 12	61 ± 11
Gender, *n*		
Male	19	9
Female	9	9
Time from Pathologic Diagnosis to Follow-up or Metastasis, days Mean ± SD	953 ± 1454	739 ± 421
AJCC Primary Tumor, *n*		
T1	0	1
T2	2	4
T3	13	7
T4	13	6
Chromosome 3, *n*		
Monosomy	14	9
Disomy	14	9
BAP1, *n*		
Positive	11	6
Negative	17	12

## Supplementary Materials

The following are available online at https://www.mdpi.com/2073-4425/11/3/271/s1, Figure S1: miRNA differentially expressed between BAP1^+/−^ and chromosome 3 disomy/monosomy uveal melanoma tumors.
