# Supplementary material for: The Potential Role of Selected miRNA in Uveal Melanoma Primary Tumors as Early Biomarkers of Disease Progression
Source: Genes (Basel). 2020 Mar 2;11(3):271. doi: 10.3390/genes11030271 (PMC7140886; doi:10.3390/genes11030271)
Supplement: Supplementary file 1 [file genes-11-00271-s001.pdf]

BAP1+ *vs* BAP1-

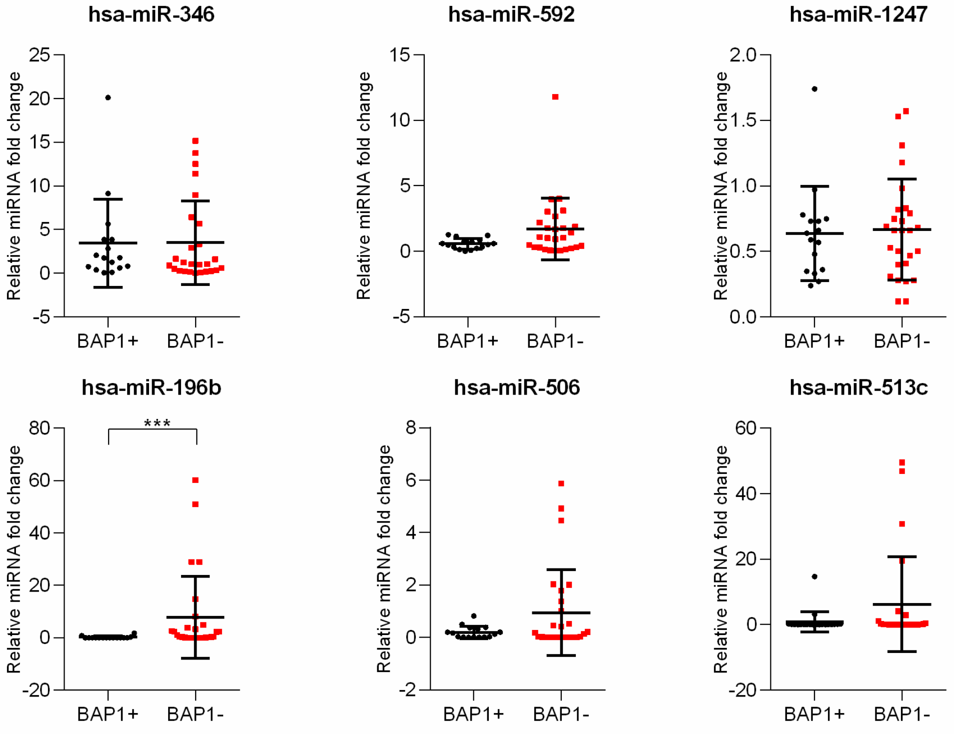

Disomy 3 *vs* Monosomy 3

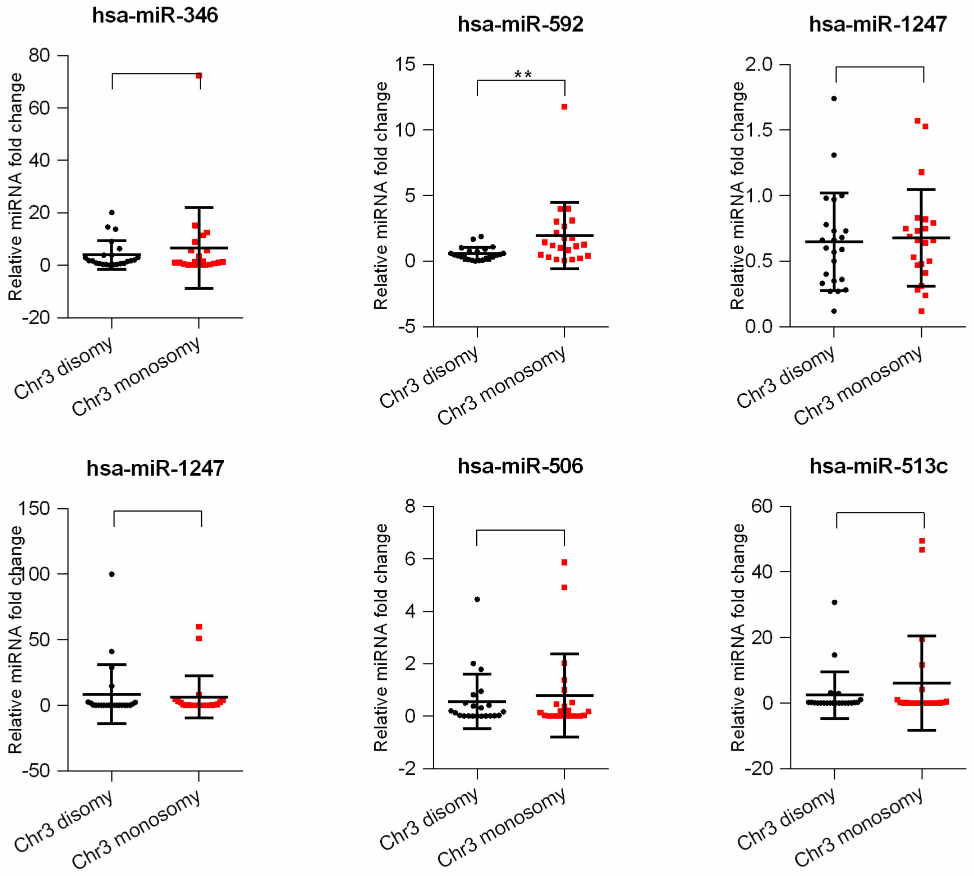

Figure S1. **miRNA differentially expressed between BAP1+/- and chromosome 3 disomy/ monosomy uveal melanoma tumors** . The results are displayed as relative miRNA fold change calculated as log2-ΔΔCt. Each dot represents an individual patient. The graph represents mean ±SD. The statistical differences between two groups were analyzed with Mann-Whitney test where: * $p < 0.05$, ** $p < 0.01$, *** $p < 0.001$, **** $p < 0.0001$.
